# Does a Common Ingroup Identity Reduce Weight Bias? Only When Weight Discrimination Is Salient

**DOI:** 10.3389/fpsyg.2019.03020

**Published:** 2020-01-21

**Authors:** Paula M. Brochu, Jillian C. Banfield, John F. Dovidio

**Affiliations:** ^1^Department of Psychology, Yale University, New Haven, CT, United States; ^2^Department of Clinical and School Psychology, Nova Southeastern University, Fort Lauderdale, FL, United States; ^3^Nova Scotia Health Authority, Halifax, NS, Canada

**Keywords:** common ingroup identity, discrimination salience, prejudice reduction, weight bias, weight discrimination

## Abstract

Compared to many other forms of social bias, weight bias is pervasive, socially accepted, and difficult to attenuate. According to the common ingroup identity model, strategies that expand group inclusiveness may promote more positive intergroup attitudes and behaviors, particularly when people are aware of unjust treatment of others included within their shared identity. Considering that most people are not aware of the social justice issue of weight discrimination, we hypothesized that a common ingroup identity would be effective in reducing weight bias primarily when unfair weight-based treatment was made salient (i.e., that fat people experience discrimination in employment). Participants were randomly assigned to conditions following a 3 (discrimination salience: weight discrimination, height discrimination, control) × 2 (group identity: common ingroup, control) design and completed an evaluative measure of weight bias. Results revealed a significant interaction, showing that when weight discrimination was salient, participants in the common ingroup identity condition reported less weight bias than participants in the group identity control condition. When a common ingroup identity was emphasized, weight bias was lower when weight discrimination was salient compared to when height discrimination was salient and the control condition in which nothing about discrimination was mentioned. These results were not moderated by participant weight. This study demonstrates that a common ingroup identity can be effective in reducing weight bias if a cue is provided that fat people experience disparate and unjust outcomes in employment. Given the serious consequences of weight bias for health and well-being, and the relative ease of implementing this prejudice-reduction intervention, the common ingroup identity model has potential application for reducing weight bias in a range of real-world settings. However, these findings should be considered preliminary until they are replicated in well-powered and pre-registered future research.

## Introduction

Weight bias includes the expression of negative attitudes, beliefs, and behaviors toward people who are higher-weight, overweight, or obese, sometimes referred to in a non-pejorative way as fat^1^ (Meadows and Daníelsdóttir, 2016; Calogero et al., 2019). Weight bias is commonly expressed, given its perceived social acceptability (Crandall et al., 2002). Indeed, regardless of their own body size, people express more negative attitudes toward fat people than they do toward a range of other stigmatized groups (e.g., Muslims, immigrants, Blacks, gay people; Latner et al., 2008; Brochu and Esses, 2011). Even though fat people are cognizant that their body size places them on the higher end of the weight continuum, fat people exhibit explicit and implicit bias, as well, toward fat people as a group (Crandall, 1994; Wang et al., 2004; Latner et al., 2008). This shared stigmatization of fat people collectively implies that even people who perceive themselves as higher-weight perceive other fat people as members of a social outgroup.

Weight bias is expressed and experienced across important domains of living in public and private, including health care, education, in the workplace, and in close relationships (Puhl and Heuer, 2009) and is thus highly consequential. Fat people report experiencing instances of weight bias almost daily in the form of verbal comments, body language and gestures, being stared at, online interactions, and physical barriers (Seacat et al., 2014; Vartanian et al., 2014). These experiences of weight bias produce negative psychological and social outcomes, ranging from eating disorders to bullying and restricted social networks (Hunger et al., 2015; Brochu et al., 2018). Moreover, the association between weight and health (e.g., increased risk of heart disease, early death) can be at least partially accounted for by the experience of weight bias (Tomiyama, 2014; Sutin et al., 2015). Accordingly, many researchers perceive a strong need to reduce weight bias (Puhl and Heuer, 2009; Daníelsdóttir et al., 2010; Lee et al., 2014). The purpose of this study was to examine whether emphasizing a common ingroup identity, a well-supported prejudice-reduction intervention in the intergroup relations literature (Gaertner and Dovidio, 2012), is effective in reducing weight bias.

Systematic reviews have noted that weight bias is difficult to reduce (Puhl and Brownell, 2003; Daníelsdóttir et al., 2010). One meta-analysis of 30 studies found that weight bias reduction interventions produced small, positive effects (Lee et al., 2014). These interventions included those that addressed weight controllability attributions, empathy and acceptance, social consensus, and incorporated other strategies (e.g., cognitive dissonance). Since these publications, some recent interventions have shown success in reducing weight bias via counterstereotypic imagined intergroup contact (Dunaev et al., 2018), psychological causal attributions (Khan et al., 2018), non-stigmatizing visual portrayals (Brochu et al., 2014), and direct intergroup contact (Koball and Carels, 2015), whereas others have produced null effects (e.g., Koball and Carels, 2015; Gloor and Puhl, 2016). Perhaps because weight bias is so strong, widespread, and often perceived to be socially acceptable, interventions that are designed to directly challenge negative weight attitudes and beliefs may arouse resistance and thus be limited in their effectiveness (Monteith et al., 1994). Instead, other techniques that aim to challenge the general psychological foundation of bias – social categorization of members of a stigmatized group as “the other” – may represent an alternative effective approach for ameliorating weight bias. The present research investigates the effectiveness of one such approach, a common ingroup identity intervention, for reducing weight bias.

The common ingroup identity model developed, in part, in an effort to explain the beneficial effects of intergroup contact on prejudice reduction, by proposing that intergroup contact helps transform people’s perceptions of their group memberships (Gaertner et al., 1996; Gaertner and Dovidio, 2005). The common ingroup identity model posits that social bias can be reduced by uniting people who belong to different social groups under one larger, superordinate group (Gaertner and Dovidio, 2000, 2009, 2012). That is, intergroup bias may be reduced by encouraging people to recategorize themselves as members of the same group (e.g., Americans, humans, college students). Creating a sense of common ingroup identity reduces bias by harnessing and redirecting the forces of ingroup favoritism (e.g., spontaneous positive evaluations and enhanced sensitivity and responsiveness to unfair treatment; Otten and Moskowitz, 2000; Tyler and Blader, 2003) to improve attitudes toward people formerly seen only as outgroup members. There is much support for the finding that a common ingroup identity reduces social bias across a range of situations (e.g., education, business, family, sports) and groups (e.g., race, ethnicity, religion, political orientation). Although no study to date has examined the common ingroup identity model within the context of weight bias, Turner et al. (2012) found that participants who recalled a nostalgic interaction with a fat person reported less negative weight attitudes, and this effect was mediated by perceptions of a common ingroup identity (i.e., that people with different weight statuses felt like members of the same group).

Creating a sense of common ingroup identity may sometimes lead people to attend less to forms of bias based on subgroup identities (e.g., race) because these identities are made less salient (Banfield and Dovidio, 2013; Dovidio et al., 2016). However, once individuals attend to this unfair treatment directed toward others previously perceived primarily as members of a social outgroup but who are now seen as members of a superordinate ingroup, people are particularly responsive to it (Tyler and Blader, 2003). Thus, a weight bias reduction intervention that emphasizes a common ingroup identity and makes disparate and potentially unjust treatment of fat people salient may be particularly effective for improving weight-based attitudes.

In the present study, we examined whether emphasizing a common ingroup identity would reduce weight bias particularly when weight discrimination was salient. Before completing an evaluative measure of weight bias, participants read information about weight discrimination in employment, height discrimination in employment, or did not read any information about employment discrimination, and then read information that emphasized a common, national ingroup identity (as Americans, fully including people of all sizes) or not (adapted from Glasford and Dovidio, 2011). Although a common ingroup identity promotes positive intergroup responding when people are aware of unjust treatment of others included within the shared identity (Banfield and Dovidio, 2013; Dovidio et al., 2016), most people consider the expression of weight bias to be relatively socially acceptable (Crandall et al., 2002; Brochu and Esses, 2011). Thus, we hypothesized that emphasizing a common ingroup identity would be effective at reducing weight bias primarily when participants were presented with information about weight discrimination (which makes weight-based unfair treatment salient). Specifically, we expected an interaction effect between discrimination salience and group identity, such that a common ingroup identity would be less effective at reducing weight bias when weight discrimination was not salient. We explored whether participant weight would moderate this effect. We measured participant weight objectively via body mass index (BMI) and subjectively via participants’ perceptions of their weight (see Blodorn et al., 2016).

## Materials and Methods

### Participants

Prior to data collection, we sought to obtain a sample size of 180 (30 participants per condition), consistent with the target sample size of 178 determined using G^∗^Power (Faul et al., 2007), specifying an alpha of 0.05, power of 0.85, and a medium effect size (*f* = 0.25). In case we had to exclude participants for incomplete or random responses, a sample of 225 participants (127 women, 97 men, 1 person who did not report gender) who lived in the United States were recruited from amazon.com’s Mechanical Turk (MTurk) service and compensated $0.50. While this compensation was on par with recommendations of others in the field (Buhrmester et al., 2011), we acknowledge that it was below the minimum wage rate of $7.25 (US) per hour, a standard that is currently commonly recommended for MTurk participants. MTurk samples are more representative and diverse than typical undergraduate samples, and typically produce high quality and reliable data (Buhrmester et al., 2011; Casler et al., 2013). Participants ranged in age from 18 to 68 years (*M* = 35.15, *SD* = 12.59). The majority of participants identified as White (*n* = 177, 79%); the remaining participants identified as Black (11%), Asian (5%), Latinx (3%), multiracial (1%), and Native American (1%).

### Materials and Procedure

After providing informed consent, participants were randomly assigned to conditions following a 3 (discrimination salience: weight discrimination, height discrimination, control) × 2 (group identity: common ingroup, control) design. First, as a manipulation of discrimination salience, participants read a passage about weight discrimination in the United States, height discrimination in the United States, or did not read anything about discrimination. The weight discrimination passage consisted of 109 words and explained that fat people experience employment discrimination (see Supplementary Material). For example, they read, “Overweight people are less likely to be hired than average weight people even when they have equivalent qualifications, skills, and experience.” The wording of the height discrimination passage was identical to the weight discrimination passage, except that it explained that short people experience employment discrimination in the United States.

Then, participants read a brief essay about weight relations in the United States that emphasized a shared identity as American, or did not read anything about weight relations. The common ingroup identity essay consisted of 278 words (see Supplementary Material) and was modeled after Glasford and Dovidio (2011). The essay explained, in part: “Regardless of whether we are average weight, overweight, or underweight, we are all first and foremost Americans…Weight should not define people…We all are a part of a common group (Americans) sharing similar values and beliefs that unite us and bring us together.”

After the manipulations, participants completed the negative judgment (e.g., “Overweight people tend toward bad behavior”), social distance (e.g., “I don’t enjoy having a conversation with an overweight person”), and attraction (e.g., “Overweight people are a turn-off”) subscales of the universal measure of bias (Latner et al., 2008).^2^ In aggregate, these evaluative subscales measure weight bias. Participants responded to each item on a seven-point Likert scale of 1 (*strongly disagree*) to 7 (*strongly agree*) and the 15 items were aggregated with higher values indicating more negative attitudes (α = 0.91).

As a manipulation check, participants completed an 11-item perceived weight discrimination measure (e.g., “Overweight people are victims of discrimination”; Brochu et al., 2011; α = 0.90) and two items about common ingroup identity perceptions (e.g., “To what extent do average weight people and overweight people feel like members of the same group?”; Turner et al., 2012; *r* = 0.52) on seven-point Likert scales. Participants then answered demographic questions that included self-reporting their weight and height, which was used to calculate BMI, and reporting their self-perceptions of their weight as *very underweight* (1), *underweight* (2), *slightly underweight* (3), *average weight* (4), *slightly overweight* (5), *overweight* (6), or *very overweight* (7). Finally, participants were debriefed about the purpose of the study and given a code to receive their compensation. The Institutional Review Board at Yale University approved all study procedures.

Participants completed the measures reported in this study as part of a larger questionnaire package. The measures reported in this study were of primary interest and were the first three measures that participants completed immediately after the manipulations. Other measures were exploratory and included to help understand, as an element of a larger project, the relationship among different aspects of weight-related orientations. All measures included in the study are presented in the Supplementary Material. All conditions and data exclusions are reported, and no other studies have been published using the data collected in the present research. No other studies have been performed that also concern the research question of this manuscript.

## Results

All participants completed all of the measures, and participants’ time spent reading the prompts was verified by reviewing the amount of time they spent on the page before submitting it. On average, participants spent 40.70 s reading the discrimination salience prompt (*SD* = 24.42) and 73.32 s reading the group identity prompt (*SD* = 44.51). Participants’ perceptions of weight discrimination varied depending on discrimination salience condition, *F*(2,219) = 2.87, *p* = 0.059, η_*p*_^2^ = 0.03, but did not vary depending on group identity condition, *F*(1,219) = 0.81, *p* = 0.369, η_*p*_^2^ = 0.004, or interact with group identity condition, *F*(2,219) = 0.88, *p* = 0.416, η_*p*_^2^ = 0.01. As expected, participants in the weight discrimination condition (*M* = 5.19, *SD* = 0.97) reported more perceived weight discrimination than did participants in the control condition (*M* = 4.84, *SD* = 1.01), *t*(150) = 2.24, *p* = 0.026, *g* = 0.35, 95% CI [−0.677, −0.036]. Participants in the height discrimination condition (*M* = 5.15, *SD* = 0.94) also perceived more weight discrimination than participants in the control condition, *t*(148) = 1.96, *p* = 0.052, *g* = 0.32, 95% CI [−0.642, 0.002], and an equivalent level of weight discrimination as did participants in the weight discrimination condition, *t*(146) = 0.26, *p* = 0.794, *g* = 0.04, 95% CI [−0.366, 0.278]. Participants’ perceptions of common ingroup identity varied depending on group identity condition, *F*(1,219) = 4.79, *p* = 0.030, η_*p*_^2^ = 0.02, but did not vary depending on discrimination salience condition, *F*(2,219) = 0.25, *p* = 0.783, η_*p*_^2^ = 0.002, or interact with discrimination salience condition, *F*(2,219) = 0.15, *p* = 0.860, η_*p*_^2^ = 0.001. As expected, participants in the common ingroup identity condition (*M* = 4.97, *SD* = 1.34) reported stronger perceptions of common ingroup identity than participants in the control condition (*M* = 4.58, *SD* = 1.41), *g* = 0.29, 95% CI [0.024, 0.549]. Thus, the manipulations of discrimination salience and group identity were orthogonal.

**FIGURE 1:**
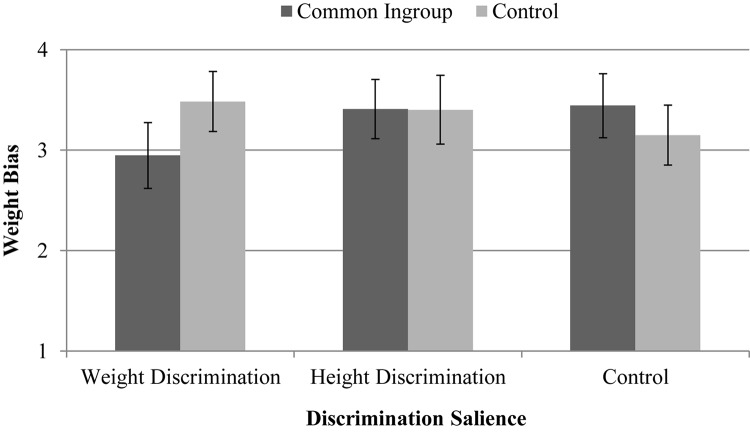
Weight bias as a function of discrimination salience and group identity. *Note.* Error bars show 95% CIs around the estimated means.

The primary hypothesis of the study, that a common ingroup identity would reduce weight bias primarily when weight discrimination is salient, is most directly tested by a 3 (discrimination salience: weight, height, control) × 2 (group identity: common, control) analysis of variance (ANOVA). In running this analysis on the evaluative measure of weight bias, neither main effect reached significance, both *F*s < 0.71, *p*s > 0.496, but as expected, the interaction between discrimination salience and group identity was significant, *F*(2,219) = 3.56, *p* = 0.030, η_*p*_^2^ = 0.03 (see Figure 1). Planned comparisons revealed that when weight discrimination was salient, weight bias was lower when a common ingroup identity was emphasized (*M* = 2.95, *SD* = 0.92) compared to the control condition in which nothing about group identity was mentioned (*M* = 3.48, *SD* = 1.03), *t*(73) = 2.39, *p* = 0.018, *g* = 0.55, 95% CI [0.084, 1.01]. Weight bias did not differ between the common ingroup identity condition (*M* = 3.41, *SD* = 1.04) and the group identity control condition (*M* = 3.40, *SD* = 0.80) when height discrimination was salient, *t*(71) = 0.02, *p* = 0.981, *g* = 0.01, 95% CI [−0.47, 0.458], or when nothing about discrimination was mentioned (common *M* = 3.44, *SD* = 1.14; control *M* = 3.15, *SD* = 0.81), *t*(75) = 1.33, *p* = 0.188, *g* = 0.30, 95% CI [−0.75, 0.151]. When a common ingroup identity was emphasized, weight bias was lower when weight discrimination was salient compared to when height discrimination was salient, *t*(74) = 2.06, *p* = 0.040, *g* = 0.47, 95% CI [0.008, 0.924], and the control condition in which nothing about discrimination was mentioned, *t*(68) = 2.14, *p* = 0.034, *g* = 0.48, 95% CI [0.002, 0.953]. No other comparisons reached statistical significance. These results replicated when participants’ BMI, perceived weight, gender, race, and age were simultaneously entered as covariates in the model. The same interaction effect and pattern of findings were found for each of the subscales of the evaluative measure of weight bias: Negative judgment, *F*(2,219) = 4.33, *p* = 0.014, η_*p*_^2^ = 0.04; social distance, *F*(2,219) = 3.49, *p* = 0.031, η_*p*_^2^ = 0.03; and attraction, *F*(2,219) = 2.97, *p* = 0.053, η_*p*_^2^ = 0.03.

In exploratory analyses, we tested the effect of (a) subjectively perceived weight or, alternatively (b) participant BMI, both as dichotomous factors (overweight vs. not overweight) and continuous predictors. Participants’ self-perceived weight ranged from 2 to 7 (*M* = 4.51, *SD* = 1.01). For subjectively perceived weight, the dichotomous factor distinguished between participants who reported that they were between “slightly overweight” and “very overweight” (*n* = 93) and those who indicated that they were between “underweight” and “average weight” (*n* = 132). Participants’ BMI ranged from 14.12 to 47.25 kg/m^2^ (*M* = 25.20, *SD* = 5.66). For the measure of BMI, participants were classified as overweight or obese (i.e., BMI ≥ 25 kg/m^2^; *n* = 96) or not overweight (i.e., BMI < 25 kg/m^2^; *n* = 125) following BMI classification guidelines (Centers for Disease Control and Prevention [CDC], 2017). BMI and self-perceived weight were highly correlated, *r* = 0.77, *p* < 0.001.

In the exploratory analyses in which subjectively perceived weight was considered as a predictor along with the manipulated variables of discrimination salience and group identity, there was a main effect of self-perceived weight on the evaluative measure of weight bias both when subjectively perceived weight was a dichotomous factor, *F* = 6.92, *p* = 0.009, and when it was treated as a continuous predictor, β = −0.15, *p* = 0.026. In both analyses, heavier participants reported less weight bias. In neither of these analyses did subjectively perceived weight moderate any effects of discrimination salience or group identity. When participant BMI was tested as a factor rather than subjectively perceived weight, there was not a significant main effect when it was dichotomized, *F* = 2.26, *p* = 0.134, but there was a significant main effect when it was tested as a continuous predictor, β = −0.14, *p* = 0.044. Higher participant BMI predicted less evaluative weight bias. Participant BMI, both when dichotomized and when treated as a continuous predictor, did not moderate the effects of the manipulated variables. Thus, participant weight status measured both subjectively and objectively did not moderate the interactive effect of discrimination salience and group identity on the expression of weight bias.

## Discussion

This study was conducted with the aim of testing the common ingroup identity model (Gaertner and Dovidio, 2012) as a weight bias reduction strategy. Results revealed that emphasizing a common ingroup identity in conjunction with making weight discrimination salient decreased weight bias relative to conditions that did not highlight a shared identity as American that included people of different weights, made height discrimination salient, or did not mention group-based discrimination. In comparison to Lee et al.’s (2014) meta-analysis of weight bias reduction interventions that found a summary effect size of *g* = 0.33, the summary effect size in this study was *g* = 0.44 (range 0.23–0.54), indicating a small–medium effect. This finding is of both practical and theoretical importance. Practically, the present work offers an important contribution to the weight bias reduction literature by introducing a novel intervention for combatting weight bias, especially considering the prevalence and harm of weight bias (Puhl and Heuer, 2009).

Theoretically, the present work extends research on the common ingroup identity model – it is, to our knowledge, the first test of the common ingroup identity model applied to weight bias – and helps to illuminate a key condition – making discrimination salient – that can moderate the effect of emphasizing common identity on social bias. It is notable that a common ingroup identity only reduced weight bias when paired with a message describing weight discrimination in employment. Previous research has revealed that making a common identity salient, while facilitating efforts to address blatant forms of unfair treatment, can sometimes have the effect of reducing perceptions that members of some subgroups encompassed within the superordinate identity may be experiencing unfair group-based treatment (Banfield and Dovidio, 2013; Dovidio et al., 2016). Because weight bias is prevalent, remains a socially acceptable form of bias (Crandall et al., 2002; Latner et al., 2008; Brochu and Esses, 2011), and is frequently justified by negative attributions to fat people (Crandall, 1994; Crandall and Eshleman, 2003), it may go largely unrecognized as unfair treatment, generally (Brochu et al., 2014). Indeed, emphasizing a common identity of people of different weights as Americans did not reduce weight bias generally; it only produced a relatively low level of weight bias when it was paired with information making the unfair treatment of fat people salient. This finding suggests the importance of highlighting the unfair treatment of people based on weight, information that can help overcome the social acceptability of weight bias (Crandall et al., 2002), and can inform understanding of weight bias as a broader social justice issue that places a common ingroup identity in context (Dach-Gruschow and Hong, 2006).

Our results further reveal, however, that gaining awareness of weight discrimination may be an integral, but not sufficient, component of weight bias reduction. Making weight discrimination salient did not reduce weight bias relative to other conditions in the absence of an emphasis on common ingroup identity. We also note that participants in the height discrimination salience condition, who also reported relatively higher levels of perceived weight discrimination, did not display a relatively lower level of weight bias even when paired with a message that emphasized a common ingroup identity. That is, even though making height discrimination salient seemed to sensitize participants to the existence of weight discrimination (at a level comparable to those who read about weight discrimination), when common ingroup identity was emphasized only those who read directly about weight discrimination (i.e., not those who read about height discrimination) showed reduced weight bias. One possible interpretation of this pattern of results is that a critical factor for reducing weight bias is an authoritative, explicit statement that negative treatment of fat people is unfair discrimination – an expression that not only makes people personally sensitive to the mistreatment but may also communicate that it is not normatively acceptable treatment. Thus, whereas people may generally be more responsive to unfair treatment of others who are regarded as ingroup (rather than outgroup) members (Tyler and Blader, 2003), because weight discrimination has been traditionally prevalent and not necessarily illegal, a strong, explicit, and authoritative statement – not simply personal awareness – may be needed to establish weight discrimination as not just unfair but also truly socially unacceptable.

Also of theoretical relevance, our results suggest the complexity of viewing weight – specifically, fat people – in terms of intergroup dynamics. Although previous research shows that fat people display both explicit and implicit biases toward fat people as a group, and seemingly do not demonstrate ingroup favoritism (Crandall, 1994; Wang et al., 2004), we found that people who perceived themselves as overweight and were heavier based on their BMI reported less weight bias than those who did not perceive themselves as overweight or were lower in BMI. It is possible that there could be some element of ingroup favoritism that mitigated, at least to some degree, the social stigmatization of fat people. However, neither subjective nor objective participant weight moderated the impact of the two manipulated variables, discrimination salience and group identity, which are directly relevant to ingroup-outgroup relations. One interpretation of this pattern of findings is that fat people do not necessarily see themselves as members of the ingroup of fat people but instead show less bias toward fat people for reasons other than ingroup favoritism, such as feelings of interpersonal similarity or more frequent social contact with other fat people. From this perspective, fat people may engage in similar group recategorization processes as thin people because they both view fat people as a social outgroup. Future research may seek to directly investigate how people self-categorize themselves with regard to their weight and identify with weight-relevant group identities while being driven by potentially competing motivations for individual distinctiveness, social identification, group belonging, and self-enhancement (Hornsey, 2008). These processes likely influence the ways in which people view themselves and others who are weight diverse, and can help illuminate the dynamics of stigmatization based on weight, the antecedents and experience of internalized weight stigma (Durso and Latner, 2008), and effective ways to reduce weight bias.

Further research is needed to test other applications and outcomes of the common ingroup identity model to weight bias. For example, a dual identity may be even more beneficial than a common ingroup identity in reducing weight bias and encouraging social change that advances weight equality (Glasford and Dovidio, 2011; Banfield and Dovidio, 2013; Ufkes et al., 2016). A dual identity allows people to retain multiple group identities by recognizing smaller subgroups within the larger common ingroup. This is reflected in a current issue of debate in the fat activism community regarding recommendations for fat people interacting with health care providers. One popular recommendation is for the fat person seeking health care services to ask their health care provider what treatment they would suggest to someone who is thin, in an effort to avoid weight discrimination (Chastain, 2013). This is akin to common ingroup identity and is referred to as weight neutrality. Others recommend that the fat person ask for treatment recommendations that are tailored for fat people, taking into account their unique weight-related needs as human beings deserving of respectful care (Aphramor, 2018). This is akin to dual identity and is known as weight inclusivity. Future research may examine the efficacy and consequences of weight-neutral vs. weight-inclusive approaches in health care interactions from the perspective of the common ingroup identity model.

Despite employing an experimental design with control conditions and a representative sample, there are a number of limitations of this study that limit its generalizability. A sensitivity power analysis conducted using G^∗^Power (Faul et al., 2007) indicated that the study had a minimal detectable effect of *f* = 0.21. In light of the small sample size limiting the ability of the study to detect small effect sizes, these data should be considered preliminary until replicated in a more highly powered and pre-registered study. It is unclear for how long attitudes change following the integrative intervention reported here. Generally, weight bias reduction interventions show limited efficacy at long-term follow-up (Daníelsdóttir et al., 2010). Longitudinal designs are needed to test the long-term efficacy of this intervention. Although the overall measure of bias employed in this study is predominantly an evaluative measure of weight bias, it contains a subscale measuring behavioral intentions (i.e., social distance; Latner et al., 2008). However, it remains unclear whether the type of attitude change found in this study would lead to behavioral changes. Future studies should investigate behavioral measures alongside measures of explicit and implicit bias that assess prejudiced attitudes, stereotyped beliefs, and affective reactions (Gaertner and Dovidio, 2012).

The present research supports the common ingroup identity model as a viable weight bias reduction strategy worthy of further research examination. The present study provides preliminary evidence for the effectiveness of a weight bias reduction intervention that emphasizes a common ingroup identity and increases the salience of weight discrimination. Given the serious consequences of weight discrimination and the relative ease of implementing this prejudice-reduction intervention, the common ingroup identity model has potential application in reducing weight bias in employment, health care, and education settings. Although these findings have important implications for intervention work and large-scale implementation, further research is needed to test the true potential of this strategy before it is applied in real world settings (Paluck and Green, 2009). Given the negative physical, psychological, and social consequences of weight bias (Brochu et al., 2018), researchers need to continue to examine the efficacy of weight bias reduction interventions. The common ingroup identity model offers insight for facilitating long-term positive social interactions that are fat accepting.

## Data Availability Statement

The raw data supporting the conclusions of this manuscript will be made available by the authors, without undue reservation, to any qualified researcher.

## Ethics Statement

The studies involving human participants were reviewed and approved by the Yale University Institutional Review Board’s Human Subjects Committee. Informed consent was obtained electronically from participants.

## Author Contributions

All authors contributed to the conception and design of the study, read and approved the submitted version. PB and JB collected the data. PB analyzed the data, and wrote and revised the manuscript. JD and JB revised the manuscript.

## Conflict of Interest

The authors declare that the research was conducted in the absence of any commercial or financial relationships that could be construed as a potential conflict of interest.

## Footnotes

1 The word “fat” is used throughout this article because it is a descriptive, non-medical term that does not refer to a normative weight standard, and its use demonstrates solidarity with fat acceptance advocacy (Crandall, 1994; Calogero et al., 2019; see also Meadows and Daníelsdóttir, 2016).

2 Participants also completed the equal rights subscale of the universal measure of bias. It was unaffected by the manipulations, all *F*s < 2.96, *p*s > 0.086, and so is not discussed further.
