# Supplementary material for: Does a Common Ingroup Identity Reduce Weight Bias? Only When Weight Discrimination Is Salient
Source: Front Psychol. 2020 Jan 21;10:3020. doi: 10.3389/fpsyg.2019.03020 (PMC6985568; doi:10.3389/fpsyg.2019.03020)
Supplement: Supplementary file 1 [file Table_1.DOCX]

Supplementary Material

# Discrimination Salience Manipulation

## Weight Discrimination Salience

Weight Discrimination in the US

Overweight people are discriminated against. Weight bias exists in the US – for example, weight discrimination has been documented at virtually every stage of the employment cycle, from hiring, promotion, and compensation to firing and disciplinary treatment. Overweight people are less likely to be hired than average weight people even when they have equivalent qualifications, skills, and experience. As a result, overweight people have higher unemployment rates than average weight people. If overweight people are employed, they earn substantially lower wages than average weight people for exactly the same work performed. Research has even demonstrated that employers evaluate an unqualified, average weight person more favorably than a qualified, overweight person.

## Height Discrimination Salience

Height Discrimination in the US

Short people are discriminated against. Height bias exists in the US – for example, height discrimination has been documented at virtually every stage of the employment cycle, from hiring, promotion, and compensation to firing and disciplinary treatment. Short people are less likely to be hired than tall people even when they have equivalent qualifications, skills, and experience. As a result, short people have higher unemployment rates than tall people. If short people are employed, they earn substantially lower wages than tall people for exactly the same work performed. Research has even demonstrated that employers evaluate an unqualified, tall person more favorably than a qualified, short person.

# Common Ingroup Identity Manipulation (modeled after Glasford & Dovidio, 2011)

Weight Relations in the US

The population in the United States is diverse in regard to weight. About 30% of the population is average weight, 34% are overweight, 34% are obese, and around 2% are underweight. People belong to different weight groups and identify with their weight (i.e., people often think about their weight throughout the course of a day). As our country becomes ever more diverse, it becomes more important that we find ways for members of different weight groups to live peacefully and productively together.

Experts from different fields have concluded that emphasizing a common category to all groups could potentially be a great asset. We are all members of a common group – American. Specifically, social scientists argue that an approach that emphasizes thinking about our common identity as Americans, without emphasizing weight differences is an essential component of long-term well-being in the United States.

Recognizing that all of us are Americans can contribute to making America a better nation. Regardless of whether we are average weight, overweight, or underweight, we are all first and foremost Americans. Instead of focusing on our particular weight identity we should celebrate that we all belong to the same big whole. Separate group identities are less relevant. In other words, while each of us is a member of a certain weight group, weight should not define people. We should have the opportunity to define ourselves and celebrate our identity as an American: We are all American!

In sum, we all are a part of a common group (Americans) sharing similar values and beliefs that unite us and bring us together. It is a common shared identity that connects all Americans.

# Additional Measures and Results

## Frequency of Weight Discrimination

Participants completed nine items indicating the extent to which they think overweight people experience discrimination in a variety of contexts (e.g., in the workforce; modified from Swim & Miller, 1999). Participants responded to each item on a scale from 0 (*never*) to 6 (*very frequently*). A 3 (discrimination salience: weight, height, control) x 2 (group identity: common, control) analysis of variance (ANOVA) indicated that participants’ perceived frequency of weight discrimination varied depending on discrimination salience condition, *F*(2, 219) = 2.94, *p* = .055, η_p_^2^ = .03, but did not vary depending on group identity condition, *F*(1, 219) = 0.36, *p* = .552, η_p_^2^ = .002, or interact with group identity condition, *F*(2, 219) = 0.32, *p* = .724, η_p_^2^ = .003. Participants in the weight discrimination condition (*M* = 4.91, *SD* = 1.04) reported more perceived frequency of weight discrimination than did participants in the control condition (*M* = 4.50, *SD* = 1.27), *t*(150) = 2.26, *p* = .025, *g* = 0.35, 95% CI [-0.673, -0.032]. Participants in the height discrimination condition (*M* = 4.86, *SD* = 0.93) also reported more perceived frequency of weight discrimination than participants in the control condition, *t*(148) = 1.86 , *p* = .064, *g* = 0.32, 95% CI [-0.642, -0.002], and an equivalent level of perceived frequency of weight discrimination as participants in the weight discrimination condition, *t*(146) = 0.37, *p* = .710, *g* = 0.05, 95% CI [-0.272, 0.373].

## Intergroup Emotions

Participants completed 12 items indicating the extent to which overweight people made them feel a variety of negative emotions (e.g., angry; Mackie, Devos, & Smith, 2000). Participants responded to each item on a scale from 1 (*not at all*) to 7 (*very much so*). A 3 (discrimination salience) x 2 (group identity) ANOVA indicated no significant main or interaction effects, all *F*s < 1.55, *p*s > .21, η_p_^2^s < .02. This nonsignificant pattern held when groups of negative emotions representing anger, anxiety, and disgust were analyzed separately.

## Threat

Participants completed eight items indicating the extent to which they thought overweight people were threatening, both realistically (e.g., Overweight people get more from this country than they contribute) and symbolically (e.g., Overweight people and average weight people have very different family values; modified from Stephan, Ybarra, & Bachman, 1999). Participants responded to each item on a 10-point Likert scale. A 3 (discrimination salience) x 2 (group identity) ANOVA indicated no significant main or interaction effects for either threat scale, all *F*s < 1.91, *p*s > .15, η_p_^2^s < .02.

## Attitude Thermometer

On an attitude thermometer, participants indicated how they felt about a range of groups, including overweight people and obese people, on a scale of 0 (*extremely unfavorable*) to 100 (*extremely favorable*; Esses, Haddock, & Zanna, 1993). A 3 (discrimination salience) x 2 (group identity) ANOVA indicated no significant main or interaction effects, all *F*s < 1.98, *p*s > .14, η_p_^2^s < .02.

## Policy Support

Participants completed three items indicating their support for laws being considered to protect people from discrimination based on weight (e.g., My state should include weight in their civil rights law in order to protect people from discrimination based on their body weight; Puhl & Heuer, 2011). Participants responded to each item on a 5-point Likert scale. A 3 (discrimination salience) x 2 (group identity) ANOVA indicated that participants’ policy support varied depending on group identity condition, *F*(1, 219) = 4.19, *p* = .044, η_p_^2^ = .02, but did not vary depending on discrimination salience condition, *F*(2, 219) = 0.37, *p* = .690, η_p_^2^ = .003, or interact with discrimination salience condition, *F*(2, 219) = 0.21, *p* = .812, η_p_^2^ = .002. Participants in the common ingroup identity condition (*M* = 3.73, *SD* = 1.11) reported more policy support than participants in the control condition (*M* = 3.44, *SD* = 1.17), *g* = 0.25, 95% CI [-0.517, 0.008].

## Moral Outrage

Participants were asked to indicate to what extent they felt angry, irritated, outraged, and disapproval (Okimoto & Brescoll, 2010; Thomas & McGarty, 2009) in reaction to learning that weight is not a protected category on a scale of 1 (*not at all*) to 7 (*very much so*). A 3 (discrimination salience) x 2 (group identity) ANOVA indicated that neither main effect reached significance, both *F*s < 1.88, *p*s > .15, but the interaction between discrimination salience and group identity was significant, *F*(2, 219) = 4.66, *p* = .010, η_p_^2^ = .04. Posthoc analyses revealed that when a common ingroup identity was emphasized, moral outrage was higher when weight discrimination was salient (*M* = 3.84, *SD* = 1.96) compared to the control condition in which nothing about discrimination was mentioned (*M* = 2.72, *SD* = 1.81), *t*(68) = 2.43, *p* = .016, *g* = .59, 95% CI [-1.073, -0.115]. When a common ingroup identity was emphasized, moral outrage was higher when height discrimination was salient (*M* = 3.77, *SD* = 2.19) compared to the control condition in which nothing about discrimination was mentioned, *t*(76) = 2.41, *p* = .017, *g* = .52, 95% CI [-0.971, -0.066]. Moral outrage did not differ between the weight and height discrimination conditions when a common ingroup identity was emphasized, *t*(74) = 0.14, *p* = .885, *g* = 0.03, 95% CI [-0.486, 0.419]. When no group identity was emphasized, moral outrage was higher when weight discrimination was salient (*M* = 3.48, *SD* = 1.98) than when height discrimination was salient (*M* = 2.59, *SD* = 1.75), *t*(70) = 1.94, *p* = .054, *g* = 0.47, 95% CI [-0.945, -0.001]. When no group identity was emphasized, moral outrage was higher in the control condition (*M* = 3.46, *SD* = 1.74) in which nothing about discrimination was mentioned compared to the height discrimination condition, *t*(70) = 1.90, *p* = .059, *g* = 0.50, 95% CI [0.025, 0.972]. Moral outrage did not differ between the weight discrimination condition and the control condition in which nothing about discrimination was mentioned when no group identity was emphasized, *t*(80) = 0.04, *p* = .966, *g* = 0.01, 95% CI [-0.444, 0.422]. When height discrimination was salient, moral outrage was higher in the common ingroup identity condition than the no group identity condition, *t*(71) = 2.60, *p* = .010, *g* = 0.59, 95% CI [-1.059, -0.112]. When no discrimination was mentioned, there was no difference in moral outrage between the common ingroup identity condition and the no group identity condition, *t*(75) = 1.67, *p* = .095, *g* = 0.42, 95% CI [-0.035, 0.87]. When weight discrimination was salient, there was no difference in moral outrage between the common ingroup identity condition and the no group identity condition, *t*(73) = 0.81, *p* = .417, *g* = 0.18, 95% CI [-0.638, 0.273].

## Social Change Motivation

Participants completed two items indicating their social change motivation (e.g., At this moment, I am extremely motivated to change policy so that weight becomes a protected category; modified from Glasford & Dovidio, 2011). Participants responded to each item on a 7-point Likert scale. A 3 (discrimination salience) x 2 (group identity) ANOVA indicated no significant main or interaction effects, all *F*s < 1.79, *p*s > .18, η_p_^2^s < .02.

## Overall Correlations between Measures

| Table S1 | | | | | | | | | |
| --- | --- | --- | --- | --- | --- | --- | --- | --- | --- |
| *Overall Correlations between Measures* | | | | | | | | | |
| Measure | 1 | 2 | 3 | 4 | 5 | 6 | 7 | 8 | 9 |
| 1. UMB | -- |  |  |  |  |  |  |  |  |
| 2. PWD | -.33*** | -- |  |  |  |  |  |  |  |
| 3. CIIP | .12 | .23*** | -- |  |  |  |  |  |  |
| 4. FWD | -.27*** | .74*** | .29*** | -- |  |  |  |  |  |
| 5. NIE | .50*** | -.13* | .15* | .02 | -- |  |  |  |  |
| 6. RST | .74*** | -.28*** | .08 | -.19** | .57*** | -- |  |  |  |
| 7. ATO | -.60*** | .11 | -.15* | .07 | -.37*** | -.52*** | -- |  |  |
| 8. PS | -.43*** | .35*** | .13* | .35*** | -.14* | -.36*** | .29*** | -- |  |
| 9. MO | -.35*** | .34*** | .07 | .35*** | -.01 | -.28*** | .30*** | .63*** | -- |
| 10. SCM | -.39*** | .29*** | .15* | .35*** | -.06 | -.26*** | .35*** | .66*** | .74*** |

*Note*. UMB = Universal Measure of Bias. PWD = Perceived Weight Discrimination. CIIP = Common Ingroup Identity Perceptions. FWD = Frequency of Weight Discrimination. NIE = Negative Intergroup Emotions. RST = Realistic and Symbolic Threat. ATO = Attitude Thermometer Overweight/Obese. PS = Policy Support. MO = Moral Outrage. SCM = Social Change Motivation.

* *p* < .05. ** *p* < .01. *** *p* < .001.

# References

Esses, V. M., Haddock, G., & Zanna, M. P. (1993). Values, stereotypes, and emotions as determinants of intergroup attitudes. In D. M. Mackie & D. L. Hamilton (Eds.), *Affect, cognition, and stereotyping: Interactive processes in group perception* (pp. 137-166). San Diego: Academic Press.

Glasford, D. E., & Dovidio, J. F. (2011). E pluribus unum: Dual identity and minority group members’ motivation to engage in contact, as well as social change. *Journal of Experimental Social Psychology*, *47*, 1021-1024. doi:10.1037/e514192012-001

Mackie, D. M., Devos, T., & Smith, E. R. (2000). Intergroup emotions: Explaining offensive action tendencies in an intergroup context. *Journal of Personality and Social Psychology*, *79*, 602-616. doi:10.1037//0022-3514.79.4.602

Okimoto, T. G., & Brescoll, V. L. (2010). The price of power: Power seeking and backlash against female politicians. *Personality and Social Psychology Bulletin*, *36*, 923-936. doi:10.1177/0146167210371949

Puhl, R. M., & Heuer, C. A. (2011). Public opinion about laws to prohibit weight discrimination in the United States. *Obesity*, *19*, 74-82. doi:10.1038/oby.2010.126

Stephan, W. G., Ybarra, O., & Bachman, G. (1999). Prejudice toward immigrants. *Journal of Applied Social Psychology*, *29*, 2221-2237. doi:10.1111/j.1559-1816.1999.tb00107.x

Swim, J. K., & Miller, D. L. (1999). White guilt: Its antecedents and consequences for attitudes toward affirmative action. *Personality and Social Psychology Bulletin*, *25*, 500-514. doi:10.1177/0146167299025004008

Thomas, E. F., & McGarty, C. A. (2009). The role of efficacy and moral outrage norms in creating the potential for international development activism through group-based interaction. *British Journal of Social Psychology*, *48*, 115-134. doi:10.1348/014466608x313774
